# Development and early‐stage evaluation of a patient portal to enhance familial communication about hereditary cancer susceptibility testing: A patient‐driven approach

**DOI:** 10.1111/hex.13702

**Published:** 2023-01-20

**Authors:** Samantha Pollard, Deirdre Weymann, Rosalie Loewen, Jennifer Nuk, Sophie Sun, Kasmintan A. Schrader, Chiquita Hessels, Dean A. Regier

**Affiliations:** ^1^ Cancer Control Research BC Cancer Vancouver British Columbia Canada; ^2^ Hereditary Cancer Program BC Cancer Vancouver British Columbia Canada; ^3^ Department of Medical Genetics University of British Columbia Vancouver British Columbia Canada; ^4^ Division of Medical Oncology, Faculty of Medicine University of British Columbia Vancouver British Columbia Canada; ^5^ Department of Medicine University of British Columbia Vancouver British Columbia Canada; ^6^ Department of Molecular Oncology BC Cancer Vancouver British Columbia Canada; ^7^ Li‐Fraumeni Syndrome Association Canada Vancouver British Columbia Canada; ^8^ School of Population and Public Health University of British Columbia Vancouver British Columbia Canada

**Keywords:** decision making, familial cancer, genetic testing, hereditary cancer syndromes, patient education

## Abstract

**Introduction:**

Genetic testing for hereditary cancer syndromes (HCSs) can improve health outcomes through cancer risk mitigation strategies. Effective communication between tested individuals and their family members is key to reducing the hereditary cancer burden. Our objective was to develop a patient portal to improve familial communication for patients undergoing HCS genetic testing, followed by an early‐phase evaluation.

**Methods:**

The portal was developed following the completion of 25 semistructured interviews with individuals having undergone HCS susceptibility testing at BC Cancer. Following initial development, we recruited patients and healthcare providers to provide critical feedback informing portal refinement. Quantitative feedback was summarized using descriptive statistics, and qualitative feedback was synthesized by two reviewers who engaged in iterative discussion within the research team to prioritize recommendations for integration.

**Results:**

The patient portal includes four key components consisting of (a) targeted educational information about hereditary cancer and HBOC syndrome associated risks and testing process overview, (b) a general frequently asked questions ‘FAQ’ page informed by the qualitative interviews, patient partner feedback, and consultation with the HCP, (c) guidance to support familial communication including a video developed with a patient partner describing their lived experience navigating the communication process and (d) a series of lay summaries of genetic test findings to support information transfer among family members. Thirteen healthcare providers and seven patients participated in user testing. Domains within which participant recommendations were provided included presentation, educational content and process clarification.

**Conclusions:**

This investigation demonstrates the value of continual integration of patient and provider preferences through the development of tools endeavouring to assist with complex genomics‐informed decision‐making. Our work aims to broaden the population‐wide impact of HCS testing programs by improving communication processes between probands and their potentially affected family members.

**Patient or Public Contribution:**

This work involved a patient partner who was actively engaged in all aspects of the research investigation including protocol development, review and editing of all study documentation (including that of the previously published qualitative investigation), interpretation of results, as well as reviewing and editing the manuscript. Patient partners and healthcare professionals were recruited as research participants to provide critical feedback on the patient portal.

## 1 INTRODUCTION

Early detection of hereditary cancer syndromes (HCSs) has the potential to reduce cancer burden through genomics‐informed prevention and treatment.^1^, ^2^, ^3^, ^4^, ^5^ HCSs, such as Hereditary Breast and Ovarian Cancer (HBOC) syndrome, Lynch syndrome, and Li‐Fraumeni syndrome explain a substantial proportion of cancer diagnoses.^3^, ^6^, ^7^ The detection of pathogenic germline variants within families commonly involves the use of multigene sequencing.^1^, ^7^ BC Cancer, the sole publicly reimbursed cancer service provider for a Canadian province (British Columbia [BC]), offers genetic testing through the Hereditary Cancer Program (HCP) for individuals with a suspected HCS (throughout BC and Yukon territory).^7^

To generate patient benefit from HCS screening programs, communication between probands (the first tested individual within a family^5^) and their biological relatives is critical.^6^, ^8^ Family members who are made aware of the presence of an HCS may choose to initiate cascade (presymptomatic) genetic testing and if indicated, undertake recommended cancer risk reduction strategies (e.g., enhanced screening frequency or prophylactic surgical interventions).

The value of interfamilial communication is not only pertinent to positive genetic test findings. In the event of negative results, biological relatives may either be reassured about their cancer risk,^9^ invited to have genetic testing themselves if they remain eligible despite their family member's results, or receive increased cancer screening recommendations based on the family history of cancer alone. For these reasons, interfamilial communication about test results and familial risk is critical to ensure accurate awareness of cancer predisposition.

Currently, in Canada, interfamilial communication is a necessary prerequisite to cascade testing and reducing concomitant disease burden. Given jurisdiction‐specific privacy laws preventing testing facilities from contacting potentially affected family members directly,^9^, ^10^, ^11^, ^12^, ^13^ it is the responsibility of tested individuals to initiate communication about results and eligibility for cascade testing with their family members. Although some jurisdictions have implemented direct contact approaches where communication about testing eligibility is led by testing facilities, such policies raise ethical and legal considerations. Some question whether a direct approach has the potential to cause undue distress among potentially affected family members or disrupt interfamilial relationships.^14^

Within jurisdictions where cascade testing is facilitated through interfamilial communication of a positive finding, it is well established that probands face a variety of challenges throughout the communication process with family members.^15^ Strained or distant relationships, limited genetic literacy, and lack of understanding about cascade testing have been identified as barriers to effective communication.^15^, ^16^, ^17^ Conversely, female gender, closer kinship, and the perception that family members will respond favourably to the information are known facilitators of familial communication and results sharing.^18^, ^19^ Due in part to known barriers to communication, cascade testing within affected families remains suboptimal.^5^, ^6^, ^17^, ^20^, ^21^ The development of strategies that respond to communication challenges necessary for ensuring the success of hereditary cancer testing programs aiming to reduce familial cancer burden.

Patient‐facing decision support tools and educational resources present an opportunity to enhance familial communication for HCS testing. Decision support tools are increasingly being developed to address informational complexity and uncertainty and to assist patients with decisions related to genetic testing, preferences for the return of results and recommendations following the return of results.^22^ For example, resources have been developed and evaluated to guide genomics‐informed reproductive decision‐making,^23^, ^24^ to clarify preferences for the return of incidental or secondary findings,^25^, ^26^, ^27^, ^28^ as well as to assist individuals deciding whether to undergo genetic testing.^29^, ^30^, ^31^ With an established need to enhance cascade testing for HCSs, interventions have been developed and evaluated specifically for *BRCA1* and *BRCA2* testing and other familial cancer syndromes.^13^ Such interventions include but are not limited to multistep skills‐building sessions with genetic counsellors, informational booklets, webinars, and motivational interviewing.^18^, ^32^, ^33^, ^34^

Building off existing evidence, we sought to engage patients and healthcare providers at BC Cancer to develop a patient portal to identify and mitigate barriers to familial communication. A primary feature of this investigation was to develop a resource for independent use by patients undergoing HCS testing at BC Cancer as an adjunct to genetic counselling appointments, for feasible implementation into standard care.

We developed and conducted user testing of a patient portal to support individuals undergoing HCS susceptibility testing at BC Cancer's HCP. For the purposes of early‐stage development and refinement within this clinical context, the patient portal was developed for individuals with suspected HBOC, receiving *BRCA1* and *BRCA2* genetic testing, who account for the largest proportion of patients referred to the HCP (approximately 80%). Given the high throughput of *BRCA1* and *BRCA2* testing within the HCP, this clinical context was determined to be appropriate within which an initial tool could be developed, evaluated, and expanded upon for feasible clinical implementation at BC Cancer.

## 2 MATERIALS AND METHODS

### 2.1 Background

The portal was developed through a multiphase, patient‐oriented approach. We first conducted 25 semistructured qualitative interviews with individuals having undergone HCS susceptibility testing at BC Cancer. Detailed methods and results of the qualitative investigation are published elsewhere.^15^ Following interviews, two qualitative analysts summarized (1) key barriers to communication, (2) recommended strategies to support patients communicating genetic testing information to their families and (3) reported preferences for the design, content and structure of a patient portal.

### 2.2 Patient portal development

Informed by the qualitative investigation, the patient portal was developed to respond to three prioritized patient portal elements, described in Table 1. Briefly, participants hoped for (1) increased support throughout the entire testing and return of results trajectory, (2) a resource for accurate information about testing, results and HCS management and (3) and multipronged, informed guidance about communicating a diagnosis of an HCS to family members. Among the latter, suggested strategies to enhance communication with family included guidance about initiating conversations with family members, as well as recommendations for managing attempts at communication perceived as unfruitful or challenging.

**Table 1:** Key portal features informed by qualitative investigation

Participant prioritized portal element	Patient portal integration	Portal page
Support throughout the pretesting, testing, return of results and communication with family trajectory	Introductory educational information to explain why testing has been recommended as well as potential benefits and outcomes Flow diagram to explain to patients the process of genetic testing Content tailored to each stage of the testing process (e.g., before testing, testing process, results, patient and familial recommendations following the return of results and communicating with family about findings)	Home
A single educational resource enhancing access to accurate information about testing, results and HCS management	Educational material (*BRCA1* and *BRCA2* genes, common terminology, population level risk estimates for individuals with and without HCS) Benefits of testing (e.g., risk reduction strategies for affected families) Links to external educational resources	Home For Patients Talking to Your Family
Guidance about communicating a diagnosis of a hereditary cancer syndrome to family members	Brief informational video presenting a patient experience communicating with family about their hereditary cancer syndrome diagnosis Written guidance about how to initiate conversations with family members Key benefits to communicating with family members about test results Email template text to facilitate written communication family members Lay summary of results for patients and their family members	Talking to Your Family Results summary pages ‘Discussing genetic testing with your family’ email template

Abbreviation: HCS, hereditary cancer syndrome.

The research team partnered with a professional web developer, located in Vancouver, British Columbia to build the online portal platform.^35^ Content was co‐developed by the multidisciplinary research team of medical oncologists, genetic counsellors, medical geneticists, health services researchers and a patient partner. The patient partner (C. H.) contributed to each aspect of the research project, including but not limited to study protocol development, critical review of study documentation, interpretation of results, portal content development, and revision and outcomes dissemination.

### 2.3 User testing instrument and development

User testing feedback was collected using an online, REDCap survey.^36^ The survey included six questions adapted from a published instrument that applied an ordinal scale to elicit critical feedback about portal features.^25^, ^37^ The survey sought feedback about clarity, length and amount of information presented, and the presentation of risks and benefits. Participants were also asked to respond to a series of open‐ended questions about key portal components (Supporting Information). Finally, developed in collaboration with genetic counsellors, HCP co‐directors and our patient partner, participants were presented with three examples of a lay summary of test results and asked to provide feedback for improvement.

### 2.4 User testing participant recruitment

Our sampling frame for user testing included patients and healthcare providers recruited from BC Cancer between February and March 2022. Eligible patient participants were members of BC Cancer's Patient and Family Experience Program, a program that invites individuals experienced with cancer to participate in research investigations in a patient partner role. Potential participants were recruited via an online newsletter. Eligible healthcare providers included medical oncologists, genetic counsellors, geneticists and those involved in clinical and leadership roles at BC Cancer's HCP. Using a convenience sampling approach, the research team generated a list of eligible healthcare providers to approach for participation, via email. Potential participants who expressed interest in providing portal feedback first completed an online consent form through REDCap. Following the provision of electronic consent, participants were then given a link to the patient portal as well as the online feedback survey, also administered using REDCap. Patient partners were provided with an honorarium of $75.00 CAD. Healthcare provider participants were not provided with an honorarium.

### 2.5 Data analysis and summary of feedback

Quantitative feedback was summarized using descriptive statistics. Qualitative feedback was reviewed by two co‐authors (S. P. and R. L.). The primary analyst (S. P.) synthesized all qualitative feedback, summarized key findings and presented results to the larger research team for discussion. Through an iterative process of review and discussion, the research team prioritized suggestions for integration into the patient portal.

## 3 RESULTS

### 3.1 Patient values integration into the patient portal

Following the completion of 25 qualitative interviews and a summary of results, investigators prioritized three key elements to inform the structure and content of the patient portal, as detailed in Table 1.

**Figure 1:**
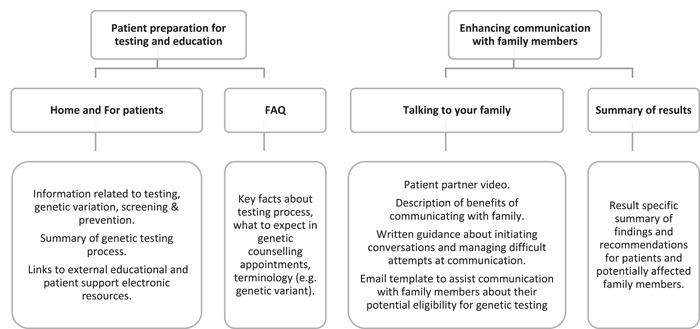
Patient portal components

Based on participant feedback that spanned educational challenges and communication barriers throughout the process of testing, the portal was designed to be provided to individuals considering HSC testing, before their pretest genetic counselling appointment. The patient portal was designed as a single website, with each major component briefly described in Figure 1.

To address participants' voiced unmet needs and preferences, the portal included six separate web pages spanning four components. The ‘Home’ page was designed to prepare patients for the testing process and their first pretesting genetic counselling appointment, to present key facts related to HCSs and provide links to each additional page. A ‘For Patients’ page provides information about *BRCA1* and *BRCA2* genes, prevention and screening strategies available to those with HBOC, as well as links to additional informational resources identified by HCP healthcare providers.

The ‘Talking to your Family’ page presents information about how and why to communicate with family, through a series of questions and answers. Questions addressed in the ‘Talking to Your Family’ page include but are not limited to, ‘Why should I talk to my family if I have received a positive (or negative) result?’, ‘How can I prepare to talk to my family?’ and ‘What if I do not feel I can speak with my family members about testing?’

Within this portal page, we also included a template email text for individuals who would prefer to broach conversations using written communication. The email template text, developed in close consultation with our patient partner and HCP healthcare providers, is intended to inform family members that the individual has undergone testing with the HCP, and a written means by which to encourage family members to contact the HCP about testing, directly. ‘Talking to Your Family’ content also addresses why and how individuals with negative or uninformative findings should consider communicating their results to their family. For example, in describing why it is important to speak with family members about a negative carrier testing result, we highlight the importance of testing for potentially at‐risk siblings who may harbour an HCS even if the proband does not.

As an additional component to the ‘Talking to your Family’ page, investigators developed a brief informational video describing one patient's lived experience communicating with their family about their HCS diagnosis. The video, approximately 4 minutes in length, describes the patient partner's experience relaying a diagnosis of an HCS to various members of her family. She describes initial and continued attempts with family members, and how she handled different reactions from relatives. Finally, she provides suggestions and recommendations, based on her own experiences, as to how to broach conversations about genetic risk using multiple approaches.

A general frequently asked questions (‘FAQ’) page provides a series of questions including additional information about testing eligibility, the testing process and where to go for more information. For example, questions addressed on the FAQ page include but are not limited to, ‘What is a genetic variant?’, ‘Can anyone have genetic testing for hereditary cancer?’ and ‘Whom can I contact if I have any questions?’

As an adjunct to the patient portal to further enhance proband education, we developed lay summaries of *BRCA1* and *BRCA2* test results. To ensure applicability to all individuals undergoing HCS testing, lay summaries were developed for both *BRCA1* and *BRCA2* carrier and index findings, including positive, negative, uninformative, and variants of unknown significance. Summaries were developed to be provided to tested individuals following discussions with their genetic counsellors about test results, to enhance understanding and facilitate conversations with family members. Summaries were embedded into the patient portal through a password‐protected healthcare provider page, wherein providers could email patients directly.

### 3.2 User testing results

A total of eight patient partners responded to the recruitment newsletter and provided informed consent via REDCap, seven of whom returned completed surveys. Thirty‐five healthcare provider email invitations were sent, 20 responded to the invitational email and indicated an interest in participating, with 14 providing informed consent and 13 healthcare providers providing survey feedback.

### 3.3 Characteristics of user testing survey participants

Among the healthcare provider sample, 5 were oncologists, and 8 were genetic counsellors, with 12/13 self‐reporting female gender. Healthcare providers reported discussing HCS a median of six times per week. All patient participants self‐reported female gender, with a median age of 58 (range: 38–70). The majority of patients reported a previous diagnosis of cancer (*n* = 6), previous genetic testing for an HCS (*n* = 4), White ethnicity (*n* = 5) and university or college education (*n* = 6). Participant characteristics are described in Table 2.

**Table 2:** Characteristics of patient and clinician participants

Healthcare providers	*N* = 13
Speciality	
Oncologist	5
Genetic counsellor	8
Female	12
How many times per week do you discuss hereditary cancer genetic testing? (median, range)	6, 0–16

Patient partners	*N* = 7
Female	7
Age (median, range)	58, 38–70
Previous cancer diagnosis	6
Type of cancer	
Breast	3
Lymphoma	1
Melanoma	1
Multiple other	1
Previous genetic testing	4
Hereditary cancer syndrome diagnosis, yes	1
Missing	1
Ethnicity	
White	5
Other	
Jewish	1
Norwegian	1
Education	
High school	1
University degree	4
College (nonuniversity)	2

### 3.4 Summary of quantitative feedback

Among quantitative survey questions, healthcare providers demonstrated broader variation in their responses regarding the presentation of information, risks and benefits, as well as amount and clarity of information, as compared to patient participants (see Table 3). Most participants reported that the way general information was presented was either good or excellent (*n* = 16/20). Similarly, most participants reported that the risk and benefits presentation, as well as the description of questions and answers, was good or excellent (*n* = 14/20 and *n* = 15/20, respectively). Fourteen (14/20) of the participants thought that the amount of information presented was just right, and all but 2 individuals (*n* = 18/20) reported that everything or most things were presented clearly. All patient participants stated that they would ‘probably’ or ‘definitely’ recommend the patient portal, with 8/13 healthcare providers stating the same.

**Table 3:** Summary of quantitative patient and healthcare provider feedback (app content)

	Patient (*n* = 7)	Clinician (*n* = 13)
The way general information was presented		
Excellent	5	3
Good	2	6
Fair	0	3
Poor	0	1
The way risks and benefits were presented		
Excellent	3	2
Good	3	6
Fair	1	5
Poor	0	0
The way questions and answers were presented		
Excellent	2	3
Good	4	6
Fair	1	4
Poor	0	0
Amount of information		
Just right	6	8
Too little	1	1
Too much	0	3
Missing	0	1
Clarity of information		
Everything clear/balanced	2	3
Most things clear	4	8
Some unclear		2
Recommend the e‐health app to others		
Definitely recommend	4	3
Probably recommend	3	5
Probably not recommend	0	4
Definitely not recommend	0	0
Missing	0	1
Preferred term to describe a genetic variant		
Gene change	0	3
Genetic variant	4	2
Genetic mutation	3	3
Genetic alteration	0	2
Gene problem	0	1
Pathogenic (if using term variant)	0	1
Abnormal result	0	1

### 3.5 Summary of qualitative feedback

All patients and 12 healthcare providers completed written qualitative comments. In general, feedback was detailed and varied. Following the analysis of the open‐ended qualitative survey questions, we identified three key categories that reflect common and comprehensively articulated suggestions, namely (a) presentation, (b) educational content and (c) process clarification, as described in detail in Table 4. Key illustrative quotes are presented in Supporting Information. Due to the fact that participant feedback related to the patient portal content and results summaries were overlapping in terms of suggested recommendations, we present an aggregated summary of qualitative feedback.

**Table 4:** Summary of patient partner and healthcare provider qualitative feedback

Domain	Key feedback
Presentation: Ensuring information is clear and easy to navigate	Use lay and neutral language throughout Present balanced risk estimates and apply multiple ways of communicating risk Present all information in a succinct and digestible format Clarify location of all educational material within the patient portal
Educational content: Information to support personal understanding and communication with family	Include additional FAQ questions to clarify testing eligibility, follow‐up process, recommendations for patients and family members following the return of results and implications of a hereditary cancer syndrome diagnosis (e.g., genetic nondiscrimination legislation) Define all technical and scientific language
Process clarification: Enhanced transparency around proband and cascade testing processes	Clearly describe HCS testing eligibility for probands and family members (according to result type and genetic family member relation) Clarify process for retesting in the future Highlight downstream benefits related to cascade testing (for relevant genetic relatives)

Abbreviation: HCS, hereditary cancer syndrome.

### 3.6 Presentation

Participants offered critical feedback about the presentation of information to improve the navigation and organization of the portal. Suggestions included the use of lay and succinct language throughout in an effort to reduce informational complexity, as well as ensure users are aware of the location of additional, more detailed educational material. Complexity and amount of scientific information were highlighted throughout qualitative responses as both necessary to ensure adequate understanding and as a potential challenge in terms of informational overload. As such, participants suggested the presentation of simplified information and phrasing, with clearly indicated links to additional, more detailed information.

Both patient and healthcare provider participants also suggested the need for neutrality in phrasing, and the careful selection of language to describe risk. For example, when presenting risk estimates (e.g., the lifetime risk of breast cancer for individuals harbouring a *BRCA1* variant), one healthcare provider participant suggested ensuring a neutral presentation of risks by presenting probabilities both positively and negatively (e.g., the probability of being diagnosed with cancer, as well as the probability of not being diagnosed with cancer over the individual's lifetime), to ensure a balanced understanding of risk. Similarly, one participant suggested softening causal language and acknowledging uncertainty related to the causal nature of pathogenic variations and the effectiveness of risk reduction strategies. In summary, participants prioritized the clear and succinct provision of accurate information, reduced informational complexity and improved ease of portal navigation.

### 3.7 Educational content

As stated, participants consistently acknowledged the complexity of information involved in HCS susceptibility testing, alongside a need to equip patients with information to make decisions about their health and their families. In addition to recommending concise language, participants also suggested the addition of information pertaining to key aspects of the testing and follow‐up process. For example, suggestions included clarifying that the evidence base around variant pathogenicity is evolving and therefore classifications have the potential to change over time, more detailed information related to available cancer screening strategies, the distinction between germline and somatic variants, further detail regarding testing eligibility, as well as detailed information about existing genetic nondiscrimination legislation.

Related to both reducing informational complexity and providing succinct educational information, participants highlighted the need to clearly define and standardize scientific terminology. There was no singular preference among participants about the appropriate terminology to describe a gene variant (Table 4). Healthcare providers varied in their preference, while most patient partners selected ‘genetic variant’ followed by ‘genetic mutation’ as their preferred term. Within the qualitative feedback survey, participants further suggested consistency of terminology through the selection of a single term to describe a genetic variant, throughout the portal.

### 3.8 Process clarification

In addition to detailed feedback regarding the user interface and educational information, participants highlighted the need to clarify current genetic testing and follow‐up processes within the patient portal. Patients and healthcare providers suggested the addition of detailed information about testing eligibility for both probands and family members, the return of results process, as well as the role genetic counsellors play in providing continued educational support following the return of sequencing results. For example, one patient participant suggested clearly presenting scenarios within which an individual would be eligible for HCP testing, such as—as the participant suggested—the presence of a strong family history of cancer alongside a current cancer diagnosis. Others wished to clarify whether it was the responsibility of the patient or the testing facility to initiate contact to determine eligibility for retesting in the future. Incorporating and clearly detailing information pertaining to the testing, diagnosis, cascade testing, and risk reduction strategy initiation process was critical, as reiterated through the qualitative feedback.

## 4 DISCUSSION

We present the development and preliminary evaluation of a patient portal to enhance communication between individuals undergoing HBOC susceptibility genetic testing and their genetic family members. Unique to this investigation is a patient and healthcare provider‐guided approach to directly address experienced communication barriers. Our work placed patient perspectives at the centre of each stage of the research process, from identifying communication challenges and recommendations for enhanced support,^15^ to portal content, design and initial evaluation. This work builds off existing investigations addressing inherent complexities associated with genomics‐informed decision‐making.^22^, ^23^, ^24^, ^29^, ^30^

User testing of patient‐facing decision support tools is an established and valued approach to ensure usability, comprehension and the capture of relevant educational information.^25^, ^38^ Through user testing, we identified a need for enhanced clarity around testing eligibility and process to enable effective communication with their relatives, while presenting educational information using approachable and concise language. Educational and process elements were raised as key to enhancing patient understanding and enabling discussions with family.

The broad and varied nature of participant recommendations suggests a need to develop flexible patient‐facing resources that are responsive to patient preferences. For example, our work demonstrates the value of generating and presenting information in a manner that acknowledges preferences for varying levels of informational detail, as well as a spectrum of approaches to navigating familial communication. Our evaluation strategy was designed to seek critical comments regarding the structure and content of the patient portal, integrate preferences, mitigate experienced challenges, and implement recommendations. Patient and healthcare provider co‐development is critical to ensure comprehensive integration of the priorities and preferences of those for whom the patient portal is intended.^25^ Findings presented through this process can be used to develop and broaden the scope of existing tools beyond *BRCA1* and *BRCA2* testing.

Previous attempts to increase interfamilial communication have demonstrated variable impacts in their ability to encourage individuals to initiate conversations and increase the uptake of cascade testing.^18^, ^20^ Published evaluations demonstrate challenges in generating patient and familial health benefits through the development of educational resources. A subsequent phase to the current investigation should consider comparative effectiveness to determine the extent to which our patient portal promotes conversations between probands and their family members, cascade testing and uptake of secondary prevention strategies among affected families. Here, we present the initial development phase, upon which additional evaluation can be investigated.

Our work can further be used to support ongoing efforts to increase cascade testing among affected families, including facilitated and direct contact of relatives by testing facilities.^5^ Frey et al. recently conducted a single‐arm feasibility study to evaluate the use of a facilitated approach, wherein testing facilities assisted probands by providing education around hereditary cancer risk, identifying potentially affected family members and communicating with relatives about cascade testing. The intervention also included genetic counselling for at‐risk relatives and distributing at‐home testing kits for those willing to undergo cascade testing. Investigators found that the facilitated approach suggested an increased uptake of cascade testing, although no comparative analyses were conducted due to the absence of a standard care control arm. Although future evaluations are required to determine whether and under which conditions facilitated and direct contact approaches are appropriate, emerging evidence demonstrates that patients may be supportive of policy shifts allowing for greater assistance from testing facilities in communicating with affected family members.^39^ As the role of facilitated and direct contact continues to be investigated cross‐jurisdictionally,^12^ educational and communication supports such as the patient portal presented here can be incorporated as an adjunct to further enhance education about HCSs, the role of cascade testing and to support communication efforts.

### 4.1 Limitations

This work should be interpreted in light of limitations. Firstly, the portal was developed and evaluated in English. For this reason, the applicability and relevance to non‐English speakers are limited and may fail to capture broader, pertinent educational and communication challenges. Addressing such disparities by translating the patient portal into additional languages relevant to jurisdiction‐specific testing populations (e.g., Chinese, French and Punjabi) and seeking critical feedback from individuals representing non‐English speaking groups will further broaden the potential impact of the tool. Of note, the patient portal does not capture information beyond the scope of *BRCA1* and *BRCA2*—associated HBOC, such as Li‐Fraumeni syndrome, Lynch syndrome or more moderate‐risk HBOC genes. The next steps in this research should seek to incorporate a breadth of HCS and elicit user feedback from affected individuals.

Second, critical feedback received during user testing may not be generalizable to the broader patient population for whom the portal was developed. The majority of participants who provided survey feedback were female, with most patient participants reporting postsecondary education and White ethnicity. Owing to study timelines, we were unable to continue recruitment efforts beyond March 2022, limiting our ability to enhance sociodemographic diversity. As a result, the spectrum of relevant information and guidance presented may not be adequately addressed in the current iteration of the portal.^40^

Our limitations speak predominantly to the ability of the patient portal to serve as a valued and effective resource, relevant to a diverse patient population. Broadening the reach of patient resources seeking to increase awareness about genetic testing and promote familial testing for potentially affected individuals will further support the mitigation of known healthcare inequities. Existing evidence suggests that multiple patient characteristics are associated with awareness of genetic testing and the uptake of publicly reimbursed healthcare services. Such factors include but are not limited to gender, language, ethnicity, income and education.^4^, ^41^, ^42^ Further efforts to ascertain critical feedback and ensure relevance to individuals representing a diversity of perspectives are warranted.

## 5 CONCLUSIONS

We present the development and preliminary evaluation of a patient portal to facilitate familial communication about HCS susceptibility testing. This portal was designed to enhance genetic literacy and equip tested individuals with guidance and resources for communicating hereditary cancer risk with their families. Engaging patients and healthcare providers at each stage of portal development as both participants and research team members has ensured a responsive approach to addressing voiced unmet needs and communication barriers within the patient portal. As a critical first step to enhancing the impacts of population base screening programmes, this work presents a patient‐guided evaluation to guide efforts to improve familial communication for patients undergoing HCS testing.

## AUTHOR CONTRIBUTIONS

**Samantha Pollard**: Conceptualization; data curation; formal analysis; investigation; methodology; project administration; writing – original draft; writing – review & editing. **Deirdre Weymann**: Conceptualization; investigation; methodology; writing – review & editing. **Rosalie Loewen**: Data curation; formal analysis; project administration; writing – review & editing. **Jennifer Nuk**: Investigation; methodology; writing – review & editing. **Sophie Sun**: Funding acquisition; investigation; methodology; writing – review & editing. **Kasmintan A. Schrader**: Funding acquisition; investigation; methodology; writing – review & editing. **Chiquita Hessels**: Conceptualization; investigation; validation; writing – review & editing. **Dean A. Regier**: Conceptualization; data curation; formal analysis; funding acquisition; investigation; methodology; resources; software; supervision; validation; writing – review & editing. Samantha Pollard had full access to all the data in the study and takes responsibility for the integrity of the data and the accuracy of the data analysis. All of the authors gave final approval of this version to be published and agreed to be accountable for all aspects of the work in ensuring that questions related to the accuracy or integrity of any part of the work are appropriately investigated and resolved.

## CONFLICTS OF INTEREST

S. P. and D. W. co‐direct IMPRINT Research Consulting Ltd. and have received payments from Roche and AstraZeneca, unrelated to the current investigation. The remaining authors declare no conflict of interest.

## ETHICS STATEMENT

The study was approved by the University of British Columbia and the BC Cancer Behavioural Ethics Board (H18‐00644). Informed consent was obtained from all subjects involved in the study.

## Supporting information

Supporting information.

## Data Availability

Link to the patient portal will be made available upon reasonable request to the corresponding author.
