# Supplementary material for: Development and early‐stage evaluation of a patient portal to enhance familial communication about hereditary cancer susceptibility testing: A patient‐driven approach
Source: Health Expect. 2023 Jan 20;26(2):774–84. doi: 10.1111/hex.13702 (PMC10010078; doi:10.1111/hex.13702)
Supplement: Supplementary file 1 — Supporting information. [file HEX-26--s001.docx]

**Supplementary file**

**S1: Qualitative feedback survey questions**

1. Do you have any suggestions about how to improve the communication of risk and probabilities within the app?
2. Can you suggest additional information related to *BRCA1* and *BRCA2* or HBOC that should be included in the For Patients page?
3. Can you suggest additional questions that should be included in the FAQ page?
4. Please use the space provided for any additional comments you have to help us improve any aspect of the app (e.g. content, length, clarity or visuals).
5. Please provide any additional comments you have to help us improve the BRCA1 VUS results summary.
6. Please provide any additional comments you have to help us improve the BRCA1 carrier positive results summary.
7. Please provide any additional comments you have to help us improve the BRCA1 carrier negative results summary.

**S2: Illustrative qualitative feedback quotes**

| Domain | Example quote |
| --- | --- |
| Presentation | *“I think that putting specific questions (what/who is this app for? What information can I find here, What can I expect? Etc.) on the home page could make it easier for patients to navigate the information.” [Healthcare provider (HCP) #12]*  *“As a young cancer patient we are always worried about what we did to “get” cancer, and that wording sounds very definitive and can cause a lot of anxiety about us somehow causing our cancer – regardless of how healthy we were prior to diagnosis.” [Patient (P) #1]* |
| Education | *“… when patients are coming to the website they are looking for detailed information on the various mutations, and what it means and what to expect from the process” (P7)*  *“I don’t know if you can ever have enough information about the risk and probabilities. It is something that all patients have a million questions about...” (P3)*  *“Numbers and probabilities are best presented with the background context as reference, and then framed in the positive and the negative (e.g., 1/10 get cancer meaning 9/10 do not)” (HCP14)* |
| Process | *“I have wondered if a new genetic mutation is discovered, will I be contacted … to check for further mutations. I am concerned that should I have more than one mutation, and family members are not […] positive could still have another mutation that has not been identified yet.” (P5)*  *‘It is important to know WHO to talk to. It is not just siblings but other blood relatives, such as aunts, uncles, nieces, nephews, and cousins. Aldo adding in something along the lines of “Your counselor/doctor can help you figure out who in your family may be at risk of having the variant”.’ (HCP15)* |
|  |  |
